# Only a Baby Tooth—Abstract

**Published:** 1898-09

**Authors:** Will H. Hall

**Affiliations:** Denver, Colorado


### Only a Baby Tooth—Abstract.

BY WILL H. HALL, DENVER, COLORADO.

Reference is here made only to the deciduous or temporary teeth as a class.

Interest in the subject which furnishes the topic of this practical treatise antedates considerably the discovery of the *first* baby's *first* tooth by the proud new parents.

Its importance does not begin nor end here, nor is it confined within the limits of baby's and childhood's tender years, as we expect to prove. An extended theoretical discussion of the proper treatment and best material for filling roots and crown of a devitalized molar would come as far short of interesting our medical brethren as would a learned paper on typhoid fever and its treatment the dental section of this assembly. Therefore, this homely topic shall be given a plain, practical but limited consideration.

"Only a baby's tooth" is expressive of insignificance, but not in that sense do we use it here. It is sometimes given professional utterance in just that sentiment, however, but no one in this body of our profession's representatives was ever guilty. The culprit lives and practices over in the next county and never attends a meeting of the City, County, State or National Medical or Dental Society. He stays at home, hoping to capture patients, while the "up-to-date" brother attends the convention feasts. He has no need of the society, and *vice versa*. Did you never hear of his saying, "Oh, its only a baby tooth. We'll pull it out, and some day a new tooth will grow in and take its place." In my imagination,

I can almost see the dim outlines of a gallows rising to receive such criminals in practice.

I would not wish to convey the idea that I think temporary teeth should never be extracted. Not extraction, but premature extraction, is the criminal act. I rather enjoyed the enviable reputation gained in my practice in Ohio of "never extracting children's teeth." This, however, was the frequent parental decision, but in practice was not adhered to when judgment dictated otherwise.

It always takes more time to explain to parent or accompanying guardian why an aching tooth should not be at once removed, than to perform a dozen such surgical operations with the apparent result, after all, that the effort was "Love's labor lost." The impression which prevails to a great extent among parents that the temporary teeth have no roots and should be extracted when they ache, because a new one will come in after a while, anyhow, is an error which should be corrected as speedily as possible. The mission of the physician and dentist is not of a remedial character alone, but prophylactic and educational also. The value of the temporary organ and its important office during that comparatively short period—the few years of child life—should be an oft-repeated lesson, until parents are impressed with their responsibility and that the period of first dentition, the care and attention necessary for their preservation until nature's physiological process accomplishes their removal, is one of great importance.

A neglected, abused or maltreated first dentition often causes a sad disfigurement of the permanent set. A premature removal of the temporary teeth is a more frequent cause of irregularity in the permanent set than a tardy removal. This is a valuable lesson the dental profession is teaching, and we are persuaded that it is not altogether in vain. Because a temporary tooth aches, it must not be concluded that extraction is the only treatment, nor simply because its "only a baby tooth," that its not worth while to give it any other consideration. True, it is much easier for the operator to extract it than to give it treatment, and parents think only of such prompt and heroic measures as will end the ache at once and forever. The family physician is often sought in such cases and yields to the desire for extraction, thereby doing violence, perhaps, to his conscience and better judgment.

The consciencious dentist (and physician) will not only protest against premature extraction, but will follow his protest with a kind and intelligent explanation of the proper treatment, with good reasons therefor, and then, if necessary, positively decline to be a party to the "slaughter of the innocents."

The preservation of the temporary teeth is important for several reasons:

*First.* They are needed for masticating purposes and must be kept in good condition for the proper performance of that function. If badly decayed, mastication cannot be properly performed, indigestion and dyspepsia result, with consequent general systemic disorder.

*Second.* They contaminate the breath, and this fetor enters the lungs; the vitiated saliva, mixed with the food, passes into the stomach; such conditions we contemplate with disgust and horror.

*Third.* Decay involves the pulp, causing pain and suffering to the child, with more or less general nervous disturbance; finally death of the pulp, resulting sooner or later in suppuration, adding another objectionable element to the contents of the stomach. The death of the pulp portends further trouble in the future, because the physiological process of absorption of the roots ceases, and these roots, minus crowns, sometimes remain for several years deflecting the permanent teeth from their course, or crowding them from their position in the arch.

*Fourth.* The temporary teeth retain the fullness of the alveolar process and maxillar development.

*Fifth.* They are needed as wedges to support and retain in its own place each contiguous tooth until the approach of the teeth of replacement.

The evils of a premature extraction of the temporary teeth are:

*First.* A possible irregularity of the permanent teeth.

*Second.* Absorption of the alveolar process and consequent contracted condition of the maxilla by reason of arrested development, thus permitting the contiguous teeth to move toward one another, closing the space, resulting in dis-allignment, impaction, or prevention of eruption of the permanent tooth. Especially important is the second or posterior temporary molar in retaining the first permanent molar in its place, and also the temporary cuspids in retaining the permanent bicuspids in their places. The premature loss of the temporary cuspid is, perhaps, the more frequent cause of disfigurement, and a more serious irregularity than any other with which the dentist has any experience.

*Finally.* Premature extraction may cause the destruction of the germ of the permanent tooth. For example, the pulps of the *first* and *second* bicuspids are formed about the fifth or sixth year, so a removal of the anterior or posterior molars at this period may result in the serious injury of the permanent tooth.

What, then, shall be done for the children, and their deciduous teeth? No single, definite plan or course can be pursued and attain the object sought, viz: the preservation of these teeth until the proper time for their removal by nature's process.

Several things are essential, viz: to assist weak nature if her resources are impoverished, restrain her if she seems violent, and correct her if her course be erratic. Hygienic science is well recognized as an essential factor in the health of the body, and dental hygiene is the key which will unlock the door to the temple of health science for the benefit of baby's "first teeth." Without this aid the man of dental science is waging a stubborn and endless battle, in the effort to *preserve* what might have been *prevented.* It is not by good guessing that our profession has arrived at an understanding of these things, but by the sure findings of scientific investigation. Our thought is not so much of hygienic influences and the benefit to these dental organs after their "line up" in the mouth, but of the importance and value of such treatment during the periods of gestation and lactation and later periods of development. It is not my purpose to consider in detail that strange and interesting operation in the human economy—the process of the development of the teeth; but when we know that the evidence of a germ, which eventually becomes a tooth, has been observed as early as the sixth week of embryonic life, with a knowledge also of the various stages and changes during the periods of development, we may reasonably expect that the influence of hygienic treatment, during those periods, will be manifested in a better quality of tooth structure. The papillæ or dental germs of the temporary teeth appear, as reported by various authorities, about as follows: The anterior molars sixth to seventh week of embryonic life, followed by the cuspids, incisors and posterior molars up to the tenth week.

The eruption of these teeth occur as follows, beginning with the incisors at about seven (7) months of age, continuing with the entire set until the twenty-fourth (24) month, when the posterior molars appear.

Therefore, from the sixth week of embryonic life, when the dental germ is first discovered, until past two years of age, when the last temporary molar is in its place, embraces a period of about three years, during which time the temporary teeth are undergoing the developing and calcifying or dentinifying process. Any diseased condition of the system which disturbs nutrition affecting or preventing the assimilation and appropriation of the lime salts, those elements which build the osseous system, will produce manifest defects in those tissues. As we nourish the body, so we can nourish the teeth. Therefore, let parents, physicians and dentists take more interest in this subject and study for the preservation of the temporary teeth by a hygienic regime, which tends toward the prevention of a low grade of tooth structure.

Here the physician's opportunity exceeds that of the dentist, but when the time and opportunity for the practice of these principles has passed, so far as benefit to the temporary teeth is concerned, then as cavities of decay appear, they should be filled.

It may be necessary to perform this operation as early as two or three years of age, but parents should understand the wisdom and value of this procedure. The selection of the kind of material for filling is governed by circumstances, but oxyphosphate of zinc or tin are generally preferred.

In conclusion, we hope that an appreciative public will more and more come to understand that not every practitioner of dental science and art is living simply to "pull" and "plug" teeth.

